# Dynamic Mobilization Exercises Improve Activity and Stride Parameters Measured with Accelerometry in Sedentary Horses

**DOI:** 10.3390/ani15202943

**Published:** 2025-10-10

**Authors:** Aritz Saitua, Joaquín Pérez-Umbría, Karelhia García-Álamo, Ana Muñoz

**Affiliations:** 1 Department of Animal Medicine and Surgery, School of Veterinary Medicine, University of Córdoba, 14071 Córdoba, Spain; aritz_sp91@hotmail.com; 2 Equine Sport Medicine Center, CEMEDE, School of Veterinary Medicine, University of Cordoba, 14071 Córdoba, Spain; joaperum@gmail.com (J.P.-U.); karegaral@gmail.com (K.G.-Á.); 3 Ekhine-Veterinary Sports Medicine, 29004 Málaga, Spain

**Keywords:** accelerometry, dynamic mobilization exercises, horse, performance

## Abstract

Dynamic mobilization exercises (DME) are an effective strategy to prevent musculoskeletal injuries and promote back health in sport horses. Previous studies focused mainly on multifidus muscle cross-sectional area, with limited data on locomotion and adaptation timing. This study evaluated locomotor changes using accelerometry, over 8 weeks of DME application in 14 sedentary horses: a DME group (*n* = 8) performing 10 different DME (3 neck flexions, 1 neck extension and 3 lateral bending exercises to each side), 5 repetitions of each DME per session, 3 sessions/week, and a control group (*n* = 6), that continued with their daily routine activities without any other training. During the study period, all horses were housed in medium-sized paddocks. Accelerometric measurements were performed at walk and trot before intervention, 2 h and 24 h after a DME session, and at 2, 4, 6, and 8 weeks. The DME group showed significant increases in dorsoventral displacement and dorsoventral and mediolateral activities from week 4, at both walk and trot, which then stabilized. Longitudinal activity increased from week 2 on trot and from week 4 at walk. Locomotor symmetry and stride length improved at week 6, while stride frequency decreased at week 8; velocity remained unchanged. These findings indicate that DME enhances dorsoventral, mediolateral and longitudinal activities, producing longer, more symmetrical strides. Overall, DME appears to promote more symmetrical movement patterns.

## Simple Summary

Maintaining a healthy back is essential for sport horses to perform well and avoid injuries, but little is known about how specific exercises affect the horse’s movement over time. This study looked at the effects of daily guided dynamic mobilization exercises (DME), on locomotion of the horses at walk and at trot, over eight weeks. Fourteen horses were included, with eight of them performing the exercises three times a week (group DME) and six continuing their usual routine used as a control (group CON). Horses that performed DME showed increased movement in the up-and-down (dorsoventral) and side-to-side (mediolateral) directions, as demonstrated by activities measured with accelerometry, with these changes appearing after 2–4 weeks, while strides became longer, more symmetrical, and stride frequency slightly slower, with these changes observed later, after 6–8 weeks. These findings show that regular guided DME can improve flexibility, elasticity, strength, and coordination of a horse. This information can help trainers, riders, and veterinarians design training programs that protect horses from injuries and improve their overall well-being and performance.

## 1. Introduction

It has been reported that motor control exercise programs reduce recurrence of back pain in humans [1]. Because back pain is also a concern in sport horses, Clayton et al. [2] and Stubbs et al. [3] developed dynamic mobilization exercises (DME) aiming to activate the multifidus muscles, which play a key role in intervertebral joint stability. Multifidus dysfunction has been associated with atrophy and joint instability in humans [4] and animals [4], which may predispose to pathological changes such as osteoarthritis [3]. Interventions that prevent or reverse this atrophy could therefore contribute to maintaining spinal health and reducing recurrence of back pain. Indeed, DMEs have been shown to increase the cross-sectional area of the multifidus in healthy, non-ridden horses after three months of regular practice [2,3].

Since these initial reports, studies have evaluated the effects of DME on thoracolumbar posture [5], multifidus morphology compared with neuromuscular electrical stimulation [6], and hindlimb muscle activity [7]. However, their contribution to locomotor pattern development in horses has not been systematically explored. De Oliveira et al. [8] measured the stride length (SL) at walk in three groups of horses: a control group (sedentary horses without additional physical activity), a group subjected to DME, and a third group, in which the researchers conducted both DME and additional gymnastic exercises, including pelvic tilting, backing, turning in small circles, and walking over a raised rail, to strengthen the abdominal and pelvic stabilizer muscles. SL increased significantly in this third group, but was unaffected in the second group, which only performed DME. In this way, when comparing the values of SL obtained before and after the 3 months of study, a difference of 0.67 ± 1.52, 2.33 ± 0.58 and 10.67 ± 2.08 cm was obtained respectively for the three aforementioned horse groups [8]. In this paper, only the SL at a walk was evaluated, arguing that it is the horse’s gait most commonly used in rehabilitation. However, these changes in SL could have been greater in other gaits, such as the trot, which is the next step on rehabilitation after a variety of exercises on a walk.

The present study was designed to evaluate locomotor changes by assessing activity (total and along the three body axes: dorsoventral, lateromedial, and longitudinal), spatiotemporal parameters, and stride regularity (REG) and symmetry (SYM) with accelerometry, at defined time points during an 8-week period of DME application. To this end, a longitudinal study of locomotor changes during walk and trot was conducted, considering both short-term effects (immediately and 24 h post-intervention) and long-term effects (every two weeks over the 8-week period). Sedentary, non-ridden horses without thoracolumbar pain were included in the study, and the results were compared with those from a control group of sedentary horses with similar characteristics, maintained under identical management conditions but not subjected to DME.

Accordingly, the following hypotheses are proposed: (1) Increased dorsoventral displacement (DVD), dorsoventral activity (DVA) and mediolateral activity (MLA) will be observed in the DME group. This is based on the expected stimulation of vertebral column flexion–extension and lateroflexion induced by the prescribed exercises; (2) Enhanced longitudinal activity (LA) will be observed in horses subjected to DME, indicating greater propulsive capacity. This improvement is expected to result in SL elongation. However, according to previous studies, SL may remain unchanged at the walk [8], but could increase at higher gait velocities, particularly at the trot; and (3) Stride REG and SYM will improve following the application of DME, reflecting a more coordinated and balanced gait pattern.

## 2. Materials and Methods

### 2.1. Horses

A total of fourteen adult horses were enrolled in the study, comprising thirteen Purebred Spanish Horses and one Anglo-Arabian, of varying sexes (1 stallion, 8 geldings, and 5 mares). The horses had a mean age of 14 years (range: 7–21 years) and an average body weight of 430 ± 20 kg (range: 360–490 kg). No significant changes in body weight were observed throughout the duration of the study.

The horses included in the study had a low level of training, as they belonged to the Veterinary Teaching Hospital and the Equine Sports Medicine Center of the University of Córdoba, where they were primarily used for teaching and research purposes in the subject of equine exercise physiology. Throughout the study period, all animals were housed in medium-sized paddocks with homogeneous earthen surfaces. Apart from their routine daily activities within the 10 × 12 m paddocks (e.g., feeding and social interactions), the only additional physical activity performed was the DME protocol (DME group). This management protocol was implemented to ensure that any observed locomotor changes could be attributed specifically to the DME, serving as a preliminary step before evaluating their effects when integrated into a structured training program.

No specific selection criteria were applied for the inclusion of animals in the study. Each horse underwent a clinical and lameness examination, including an assessment of the back, to confirm the absence of clinically evident lameness or, if present, only mild lameness (≤grade 2 out of 5) according to the guidelines of the American Association of Equine Practitioners (AAEP), as well as the absence of thoracolumbar pain.

The horses were then randomly assigned into two groups: eight horses were allocated to the DME group, and six horses to the control group (CON).

### 2.2. Dynamic Mobilization Exercises

**Figure 1:**
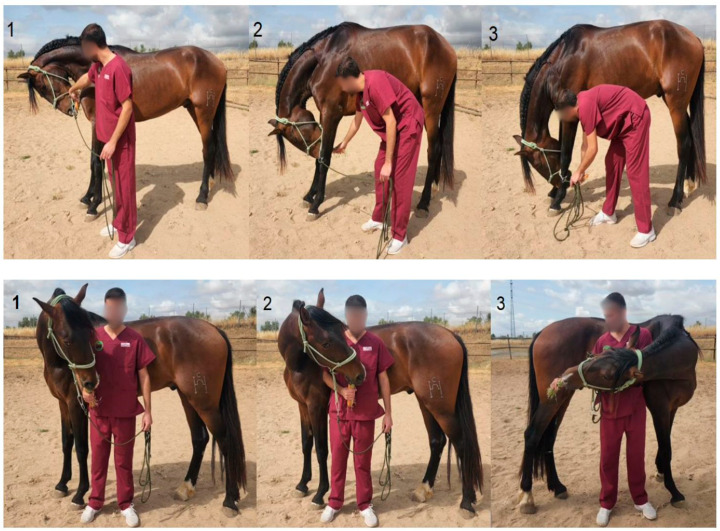
Representation of application of several dynamic mobilization exercises (DME). Above: Cervical flexion exercises, 1: chin to chest; 2: chin between carpi; 3: chin between fore fetlocks: Below: Cervical lateral bending exercises: 1: chin to girth; 2: chin to flank; 3: chin to stifle.

Horses in the DME group performed a DME protocol as previously described by Stubbs and Clayton [9]. The protocol included a total of 10 DME: three cervical flexion exercises in the sagittal plane at different positions: chin to chest, chin between the carpi, and chin between the fore fetlocks. Additionally, one neck extension exercise was performed, in which the horse was encouraged to extend the neck forward as far as possible. The protocol also comprised three lateral cervical bending exercises to each side (right and left), involving the following positions: chin to girth, chin to flank, and chin to stifle (Figure 1).

Each DME session consisted of ten different exercises, each repeated five times per session. Once the horse reached the maximum range of motion in flexion or extension, while standing in a square stance, the position was maintained for 5 s. The training protocol included three DME sessions per week over an eight-week period. All exercises were carried out by the same researcher (J.P.-U.), who had received prior training to ensure the standardized execution of the mobilizations.

Locomotor evaluations were conducted at multiple time points throughout the study using accelerometry to assess changes over time.

### 2.3. Data Collection

For the accelerometric evaluations, horses were equipped with a portable 3D gait analysis system (Equimetrix, Centaure-Metrix^®^, Evry, France), which integrates three orthogonally positioned accelerometers to measure acceleration along the three body axes. The system includes a data logger and dedicated software (Equimetrix-Centaure 3D^®^, Evry, France) for processing the acceleration signals. The accelerometer records data continuously while the horse is moving, with a sampling rate of 100 Hz.

The accelerometer was positioned on the caudal aspect of the sternum, between the right and left *pectoralis ascendens* muscles at the level of the girth, following the placement protocol recommended [10]. This location is close to the horse’s center of gravity, ensuring good device stability and providing reliable acceleration data. To maximize reproducibility, the same researcher (J.P.-U.) was responsible for positioning the accelerometer and conducting all locomotor evaluations throughout the study.

Accelerometric assessments were conducted on the same training track at multiple time points throughout the study. Data were recorded with the horses led in hand at both walk and trot over a distance of 80 m. Each horse completed at least four runs at walk and four runs at trot, ensuring a consistent velocity for each horse and gait. Accelerometric parameters were calculated from three measurements per run, resulting in a total of 12 measurements per gait (walk and trot) for each horse. The mean of these 12 measurements was used for subsequent analysis.

**Figure 2:**
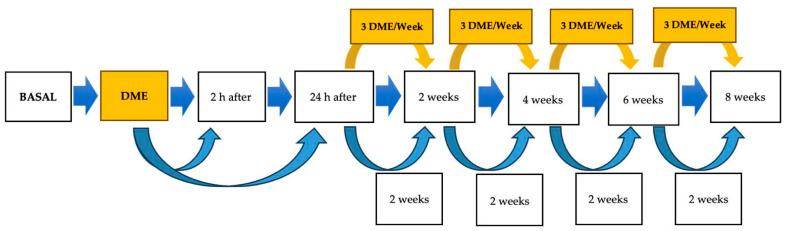
Temporal schedule of dynamic mobilization exercises (DME) and accelerometric evaluations conducted throughout the study. The CON group underwent only accelerometric evaluations without participation in the DME protocol.

To assess short-term locomotor changes associated with a single DME session, evaluations were conducted at baseline (prior to the intervention), 2 h post-session, and 24 h post-session. For long-term assessment, recordings were obtained at 2, 4, 6, and 8 weeks. Horses in the CON group underwent locomotor evaluations at identical time points but did not receive the DME intervention. The timeline of the locomotor evaluation protocol is illustrated in Figure 2.

### 2.4. Locomotor Parameters

Three groups of parameters provided by the accelerometer were included in this research: dorsoventral displacement (DVD) and activities, coordination and spatiotemporal parameters.

DVD was estimated through double integration of the dorsoventral acceleration signal. The activities analyzed included dorsoventral activity (DVA, W/kg), representing acceleration along the dorsoventral axis and therefore, elasticity; longitudinal activity (LA, W/kg), reflecting propulsion along the craniocaudal axis; and mediolateral activity (MLA, W/kg), indicating acceleration along the mediolateral axis, associated with flexibility. DVA was calculated as the integral of the power spectrum obtained via Fast Fourier Transform (FFT) of the dorsoventral acceleration signal and reflects limb suspension and loading dynamics. LA represents craniocaudal propulsion and was derived from the integral of the power spectrum of the longitudinal acceleration signal. MLA quantifies lateral activity, calculated similarly from the power spectrum of the mediolateral acceleration signal, measuring acceleration and deceleration side-to-side. The sum of these three components (DVA, LA, and MLA) constitutes the total activity (TA, W/kg).

Stride coordination parameters analyzed included stride regularity (REG) and stride symmetry (SYM), both dimensionless indices. REG quantifies the similarity of acceleration patterns across successive strides over a given time period, reflecting the consistency of the gait. SYM assesses the similarity between left and right stride acceleration patterns, indicating bilateral symmetry [10,11,12,13].

Stride spatiotemporal parameters included stride frequency (SF, strides/s or Hz) and stride length (SL, m), with velocity obtained from GPS (Polar heart monitor M430^®^, Polar Electro Oy, Kempele, Finland) measurements for each accelerometric assessment. The heart rate monitor with GPS for velocity measurement and the accelerometer were started simultaneously at the beginning of the accelerometric recordings. SF was calculated by detecting the frequency of the major peak of the power spectrum obtained via Fast Fourier Transformation of the dorsoventral acceleration signal, while SL was calculated by dividing the velocity by SF [11].

### 2.5. Statistical Analysis

Results are presented as the percentage of variation for each locomotor parameter, calculated relative to values obtained in the baseline assessment. Positive values indicate an increase from baseline, while negative values indicate a decrease relative to baseline.

The normality of the percentages of variation was assessed using the Shapiro–Wilk test. As the percentages were not normally distributed, non-parametric analyses were applied, and data are presented as median and quartiles. To account for the individual effect, each horse served as its own control, and comparisons between measurement times within each group (DME and CON) were performed using Friedman and Wilcoxon tests for repeated measures. Comparisons between groups at each measurement time were conducted using the Mann–Whitney test.

Statistical analyses were performed using Statistica v.14.0 and IBM SPSS Statistics 21. Statistical significance was set at *p* < 0.05.

## 3. Results

### 3.1. Dorsoventral Displacement and Activities

**Figure 3:**
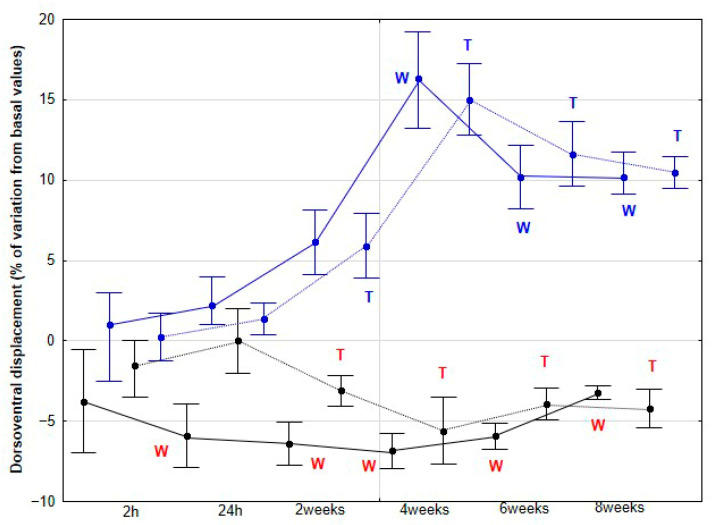
Medians and quartiles of dorsoventral displacement (DVD), expressed as a percentage of baseline values, for each evaluation time in the CON group (black lines) and the DME group (blue lines) during walking (solid lines) and trotting (dashed lines). Significant differences between each evaluation time with 2 h-time are indicated in black for the CON group and in blue for the DME group (W = walk; T = trot). Significant differences between CON and DME groups in each evaluation time are shown in red (W = walk; T = trot). Statistical significance was set at *p* < 0.05.

**Figure 4:**
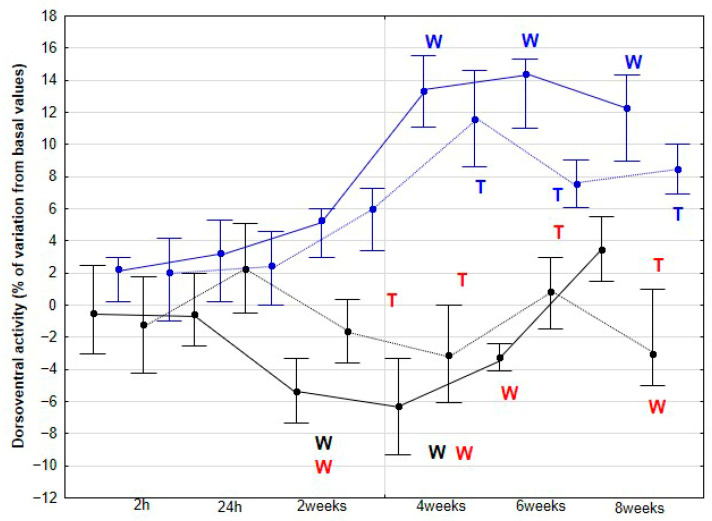
Medians and quartiles of dorsoventral activity (DVA), expressed as a percentage of baseline values, for each evaluation time in the CON group (black lines) and the DME group (blue lines) during walking (solid lines) and trotting (dashed lines). Significant differences between each evaluation time with 2 h-time are indicated in black for the CON group and in blue for the DME group (W = walk; T = trot). Significant differences between CON and DME groups in each evaluation time are shown in red (W = walk; T = trot). Statistical significance was set at *p* < 0.05.

**Figure 5:**
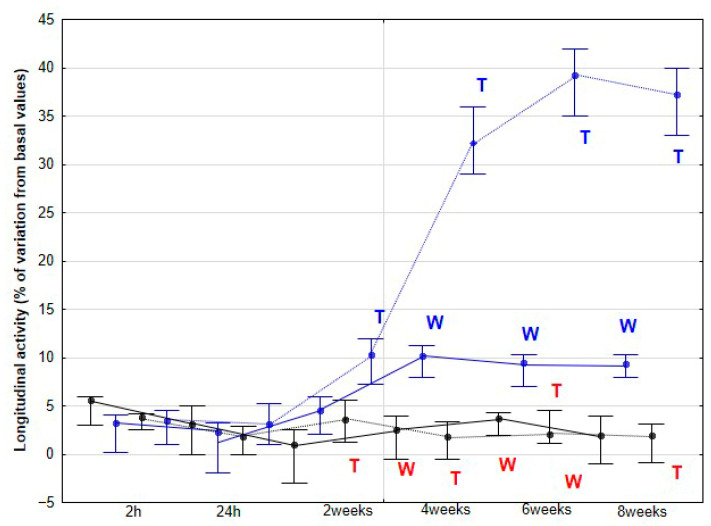
Medians and quartiles of longitudinal activity (LA), expressed as a percentage of baseline values, for each evaluation time in the CON group (black lines) and the DME group (blue lines) during walking (solid lines) and trotting (dashed lines). Significant differences between each evaluation time with 2 h-time are indicated in black for the CON group and in blue for the DME group (W = walk; T = trot). Significant differences between CON and DME groups in each evaluation time are shown in red (W = walk; T = trot). Statistical significance was set at *p* < 0.05.

**Figure 6:**
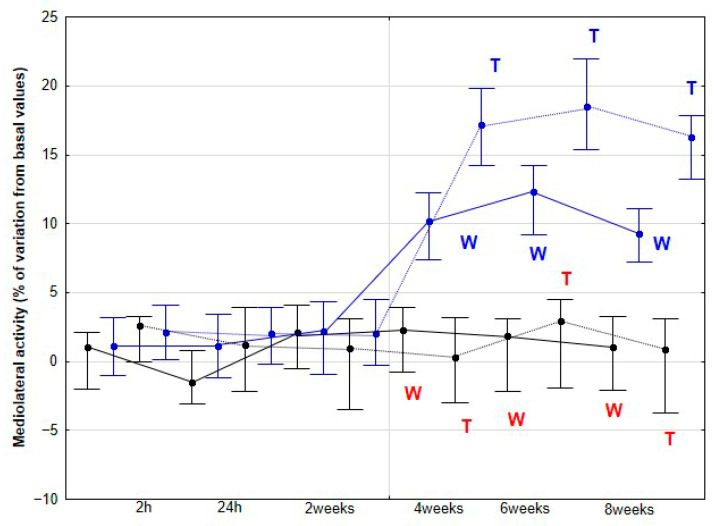
Medians and quartiles of mediolateral activity (MLA), expressed as a percentage of baseline values, for each evaluation time in the CON group (black lines) and the DME group (blue lines) during walking (solid lines) and trotting (dashed lines). Significant differences between each evaluation time with 2 h-time are indicated in black for the CON group and in blue for the DME group (W = walk; T = trot). Significant differences between CON and DME groups in each evaluation time are shown in red (W = walk; T = trot). Statistical significance was set at *p* < 0.05.

**Figure 7:**
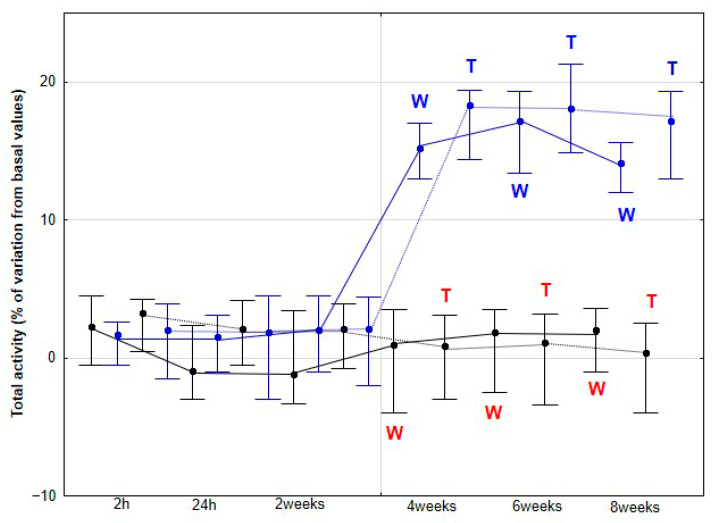
Medians and quartiles of total activity (TA), expressed as a percentage of baseline values, for each evaluation time in the CON group (black lines) and the DME group (blue lines) during walking (solid lines) and trotting (dashed lines). Significant differences between each evaluation time with 2 h-time are indicated in black for the CON group and in blue for the DME group (W = walk; T = trot). Significant differences between CON and DME groups in each evaluation time are shown in red (W = walk; T = trot). Statistical significance was set at *p* < 0.05.

The percentage variations from the baseline accelerometric evaluation values for DVD and activities are presented in Figure 3, Figure 4, Figure 5, Figure 6 and Figure 7.

The percentage variation of DVD relative to baseline values showed no significant changes in the CON group, either at the walk (*p* = 0.741) or at the trot (*p* = 0.437). In contrast, the DME group exhibited a significant increase in this percentage at 4 (*p* < 0.001), and 8 weeks during the walk (*p* < 0.001), and at 2 (*p* < 0.001), 4 (*p* < 0.001), 6 (*p* < 0.001), and 8 weeks (*p* < 0.001), during the trot, with the maximum variation observed at 4 weeks (Figure 3).

The percentage variation of DVA increased significantly in the DME group at 4 (*p* < 0.001), 6 (*p* < 0.001), and 8 weeks (*p* < 0.001), both at walk and trot. In contrast, no significant changes were observed in the CON group for either gait (*p* = 0 > 0.05). From week 2 (*p* = 0.002) onward, the DME group showed higher DVA variation percentages compared with the CON group, and this difference persisted through the final assessment at week 8 (*p* < 0.001) (Figure 4).

In the DME group, the percentage variation in LA relative to baseline increased, appearing at 4 weeks for walking (*p* < 0.001), and as early as 2 weeks for trotting (*p* = 0.001), while the CON group exhibited no significant changes (*p* > 0.05) (Figure 5).

No significant changes were observed in the percentage variation of MLA and TA relative to baseline during either walking or trotting in the CON group (*p* > 0.05). Conversely, the DME group demonstrated a significant increase in these parameters at 4 (*p* < 0.001), 6 (*p* < 0.001), and 8 weeks (*p* < 0.001), in both gaits. Furthermore, at these time points, the percentage variation of MLA and TA relative to baseline was significantly greater in the DME group compared with the CON group (*p* < 0.001) (Figure 6 and Figure 7).

### 3.2. Coordination Parameters of the Stride

**Figure 8:**
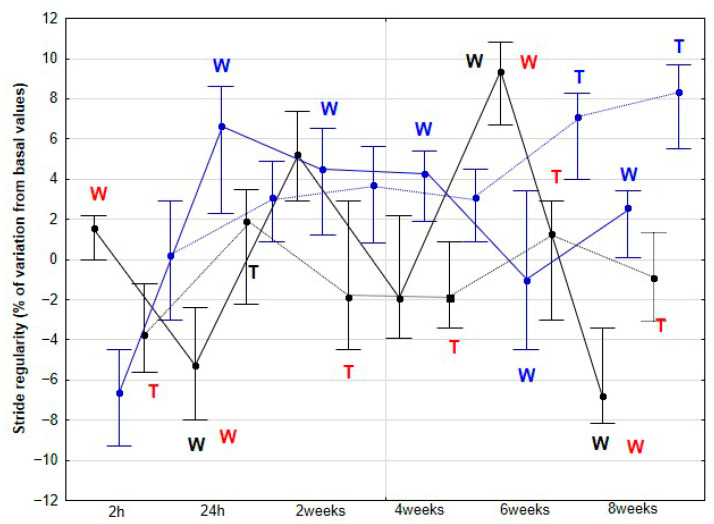
Medians and quartiles of stride regularity (REG), expressed as a percentage of baseline values, for each evaluation time in the CON group (black lines) and the DME group (blue lines) during walking (solid lines) and trotting (dashed lines). Significant differences between each evaluation time with 2 h-time are indicated in black for the CON group and in blue for the DME group (W = walk; T = trot). Significant differences between CON and DME groups in each evaluation time are shown in red (W = walk; T = trot). Statistical significance was set at *p* < 0.05.

**Figure 9:**
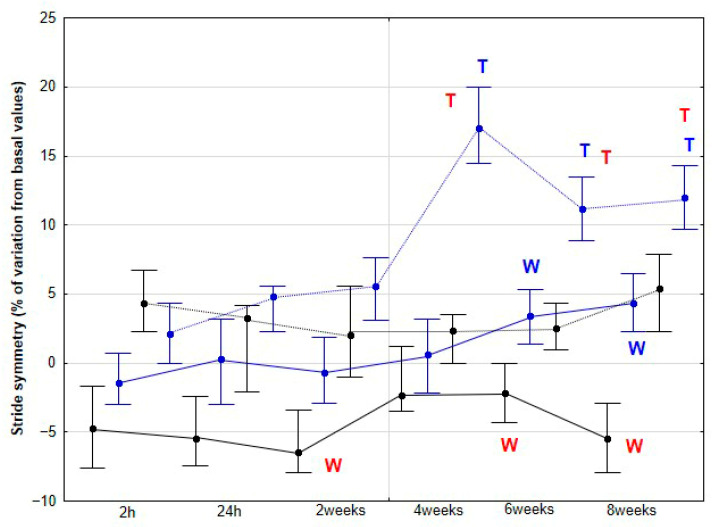
Medians and quartiles of stride symmetry (SYM), expressed as a percentage of baseline values, for each evaluation time in the CON group (black lines) and the DME group (blue lines) during walking (solid lines) and trotting (dashed lines). Significant differences between each evaluation time with 2 h-time are indicated in black for the CON group and in blue for the DME group (W = walk; T = trot). Significant differences between CON and DME groups in each evaluation time are shown in red (W = walk; T = trot). Statistical significance was set at *p* < 0.05.

The percentage variation of REG showed significant but inconsistent changes at both walk and trot in both groups of horses (Figure 8). Regarding SYM, an increase in the percentage variation was observed in the DME group at 6 (*p* = 0.021) and 8 weeks (*p* = 0.015) at walk and at 4 (*p* < 0.001), 6 (*p* < 0.001) and 8 (*p* < 0.001) weeks at trot when compared to baseline. Significant differences between DME and CON were observed at 2 (*p* = 0.031), 4 (*p* < 0.001), and 6 weeks (*p* < 0.001) during walking, and at 4 (*p* < 0.001), 6 (*p* < 0.001), and 8 weeks (*p* < 0.001) during trotting (Figure 9).

### 3.3. Spatiotemporal Parameters of the Stride

Particular care was taken to homogenize velocity by selecting runs in which the velocity was similar, since changes in this parameter can affect both the spatiotemporal parameters of the stride and activities. For this reason, no significant differences in velocity were found across the different time points or between the two groups of horses.

**Figure 10:**
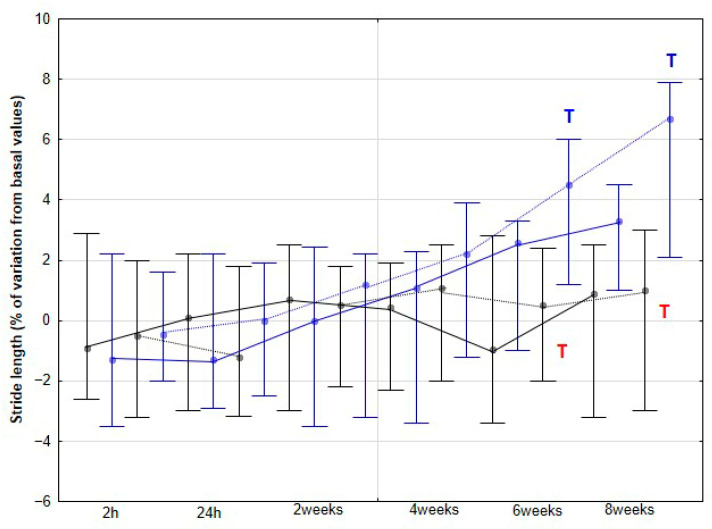
Medians and quartiles of stride length (SL) expressed as a percentage of baseline values, for each evaluation time in the CON group (black lines) and the DME group (blue lines) during walking (solid lines) and trotting (dashed lines). Significant differences between each evaluation time with 2 h-time are indicated in black for the CON group and in blue for the DME group (W = walk; T = trot). Significant differences between CON and DME groups in each evaluation time are shown in red (W = walk; T = trot). Statistical significance was set at *p* < 0.05.

**Figure 11:**
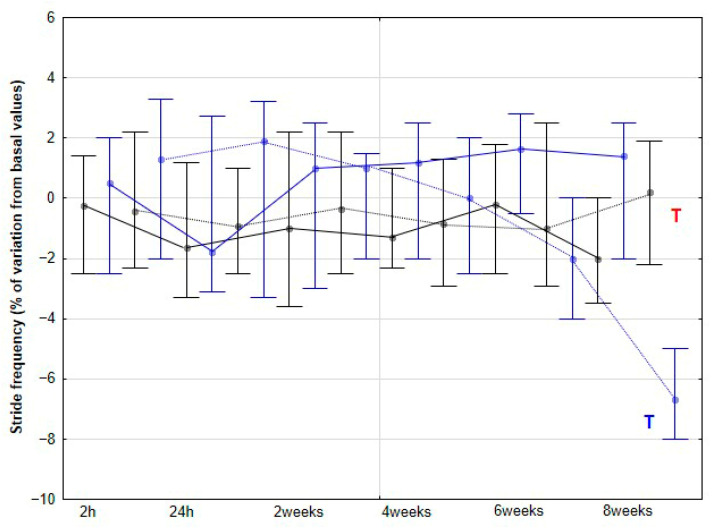
Medians and quartiles of stride frequency (SF) expressed as a percentage of baseline values, for each evaluation time in the CON group (black lines) and the DME group (blue lines) during walking (solid lines) and trotting (dashed lines). Significant differences between each evaluation time with 2 h-time are indicated in black for the CON group and in blue for the DME group (W = walk; T = trot). Significant differences between CON and DME groups in each evaluation time are shown in red (W = walk; T = trot). Statistical significance was set at *p* < 0.05.

The percentage variation of SL relative to baseline did not change significantly in the CON group at either walk or trot, nor in the DME group at walk (*p* > 0.05), even though a non-significant trend towards an increase was found at 8 weeks (*p* = 0.079). However, at trot, the DME group showed a significant increase in SL percentage variation at 6 (*p* = 0.032) and 8 weeks (*p* < 0.001), with significant differences also observed compared to the CON group at the same time points (*p* = 0.025 and *p* = 0.003 for 6 and 8 weeks respectively) (Figure 10). Regarding SF, the only significant change observed was a significant reduction in the percentage variation relative to baseline values at 8 weeks in the DME group at trot (*p* < 0.001) (Figure 11).

## 4. Discussion

The present study yielded several notable findings. Implementation of DME in healthy sedentary horses was associated with a significant increase in activity across all three orthogonal body axes, first detectable at 4 weeks and thereafter maintained without further significant change. Improvements in stride SYM were observed at a later stage, reaching statistical significance at 6 weeks. Similarly, SL showed a significant increase beginning at 6 weeks, whereas SF demonstrated a significant decrease, becoming apparent at 8 weeks. To our knowledge, this is the first study to delineate the temporal dynamics stride parameters in horses undergoing DME.

As the first hypothesis to be tested in our study, we proposed that horses subjected to DME would exhibit increased DVD, DVA and MLA, reflecting enhanced dorsoventral elasticity and lateroflexion flexibility. Our findings support this first hypothesis, as a significant increase in these three parameters was observed in the DME group from 4 weeks onwards at both walk and trot, with no further changes at 6 and 8 weeks, and no comparable variation was detected in the CON group. This plateau suggests that maximal adaptation may have been achieved in these previously sedentary horses, or that additional gains might require higher exercise frequency, extended intervention duration, or the incorporation of complementary training modalities.

The mechanisms underlying the locomotor changes observed with the application of DME remain largely unknown. Pfau et al. [14] reported an increase in DVD over the thoracolumbar region following a 4-week elastic resistance band training program. However, as these authors acknowledged, it was not possible to determine whether these changes were attributable to the elastic band itself or to the exercise protocol. Similarly, the use of the Pessoa training aid has been associated with increased mid-back DVD and lumbosacral flexion, changes proposed to result from a combination of head and neck lowering together with the activation of spinal flexor and abdominal muscles [15]. While such findings suggest that DME may exert a strengthening effect on spinal flexor and abdominal musculature, it is important to note that the underlying mechanisms remain speculative and have not yet been directly confirmed. Furthermore, although evidence from human medicine supports training-related improvements in spinal mobility with beneficial effects on postural control and performance [16], the direct extrapolation of these findings to equine models should be made with caution.

Comparable uncertainties exist in relation to spinal manipulation therapies, where Haussler et al. [17] reported increased DVD but without clarification of the mechanisms involved. Proposed explanations include changes in the viscoelastic properties of paraspinal soft tissues [18] or the stimulation of peripheral articular receptors and central nervous system pathways leading to muscle relaxation, improved motor function, and enhanced spinal flexion [19]. Although it is plausible that the DME protocol applied in the present study could induce similar effects, such an assumption remains hypothetical. The absence of direct mechanistic evidence, together with variability in methodological approaches across studies, underscores the need for controlled experimental designs that can disentangle the specific contributions of DME to spinal function.

Our second hypothesis, proposing that horses subjected to DME would exhibit greater LA as an indicator of increased propulsive capacity, with this enhancement potentially leading to SL elongation, was also supported by our results. In racing and performance contexts, LA has been consistently associated with superior athletic output. Both harness trotters and flat racing Thoroughbreds with higher activity values along the longitudinal axis demonstrate better performance outcomes [11,20,21]. Elite racehorses appear to optimize propulsion along this axis while minimizing unnecessary dorsoventral oscillations, a strategy that maximizes energy efficiency [10,11]. Beyond velocity-oriented disciplines, LA provides valuable insight into locomotor strategies under varying competitive demands. Viry et al. [22] reported that elite horse–rider dyads maintain consistent gait and propulsion patterns over long distances, whereas less experienced dyads adjust gait selection and propulsion intensity as fatigue progresses. More recently, LA has been proposed as a tool for fatigue detection. Darbandi et al. [23] identified walking stance duration and trotting limb longitudinal displacement as reliable biomechanical indicators of fatigue, with horses increasing stance duration and reducing longitudinal displacement as fatigue developed. These findings highlight that LA might be used as an indicator of adaptive locomotor strategies.

In our study, LA exhibited a small but significant increase at 2 weeks, followed by a more pronounced rise at 4 weeks, after which it remained statistically stable at the trot. At the walk, a significant increase was observed later, at 4 weeks. Considering the previously discussed role of LA in equine athletic performance, it is plausible to speculate that the observed LA enhancements resulting from DME implementation could translate into improved sporting performance. However, this aspect was not directly assessed in the present study. Nonetheless, these findings provide a strong rationale for future investigations specifically designed to evaluate the potential impact of DME on competitive performance outcomes. In humans, a variety of studies—ranging from patients with low back pain to athletes undergoing performance training [24,25,26]—have demonstrated that improvements in core function and related performance parameters can emerge after approximately 4 weeks of intervention. This timeframe corresponds to the period in which we observed the most significant changes in our study in horses subjected to DME, whereas no changes were detected in the CON group.

The changes in activities observed in our study were accompanied by an increase in SL, but only at the trot and, notably, from 6 weeks onward. This increase may be a consequence of the prior enhancement in LA, reflecting greater propulsive forces. No significant changes were detected at the walk, although a non-significant trend toward an increase was observed at 8 weeks. De Oliveira et al. [8] found no variations in SL at the walk in horses subjected to DME, whereas horses performing additional gymnastic exercises exhibited a significant increase in SL. These authors associated the increase in SL after three months of gymnastic exercises with greater flexion in the proximal limb joints, suggesting that both spinal and limb kinematic changes contributed to the observed stride elongation. In our study, we did not collect specific kinematic data for the back or limbs, preventing a direct assessment of these mechanisms. However, the earlier increase in LA from 4 weeks, which can be interpreted as greater force generation along the longitudinal axis, together with enhanced MLA from the same time point, likely facilitated stride elongation. Notably, this effect on SL appeared later than the initial locomotor changes and was only evident at trot. Taken together, these results might indicate that DME induces progressive biomechanical adaptations in horses, beginning with increased propulsive activity and medio-lateral flexibility.

Whether these adaptations precede or occur concomitantly with changes in the size of the *multifidus* muscles remains unknown. This represents a limitation of our study, as no measurements of *multifidus* cross-sectional area were performed. To fully characterize the temporal profile of locomotor adaptations, it would have been highly desirable to include ultrasonographic assessments of muscle cross-sectional area. Unfortunately, these measurements could not be conducted in the present investigation, constituting an important limitation that should be addressed in future studies. Furthermore, integrating these accelerometric studies with kinematic limb assessments and electromyography could provide a more comprehensive understanding of the effects of DME application in horses. Additionally, future studies could include muscle biopsies, particularly of the multifidus or other key stabilizer muscles, to investigate histological, immunohistochemical, and molecular changes, providing insights into underlying mechanisms and potential therapeutic targets.

Our third hypothesis was only partially fulfilled, as the changes observed in stride REG throughout the study were erratic and difficult to interpret, with no clear justification. However, the expectation of finding a more balanced pattern, with an improvement in stride SYM, was met, although this change appeared later in the study compared to that observed in the accelerometric activities. Previously, de Oliveira et al. [27] reported an increase in locomotor REG and SYM in horses subjected to gymnastic training, which included several types of DME as well as other activities, such as stepping over a raised pole and backward steps. Therefore, our results partially align with those of the aforementioned authors. However, in their study, locomotor assessments were performed only at the beginning and at the end of the 3-month training period. De Oliveira et al. [27] argued that these changes reflected an improvement in the overall quality of the horse’s movement.

In addition to the lack of measurement of the cross-sectional area of the multifidus muscles, as previously mentioned, our study has several limitations. The number of horses was limited, and they were sedentary animals; therefore, the interaction between DME and training could not be evaluated. Second, the study duration was only 8 weeks. It would be of interest to assess performance variations and locomotor pattern changes induced by DME in actively competing horses, as well as its potential protective effect on the incidence of musculoskeletal injuries. Furthermore, due to logistical reasons, the number of horses included in the CON and DME groups was different. Finally, an individual effect on our results cannot be ruled out. Nevertheless, regarding this last point, although the magnitude of the changes varied, the locomotor changes detected followed the same direction in all the studied horses

## 5. Conclusions

Our results demonstrate that the application of DME in sedentary horses, free from thoracolumbar pain and lameness, induces significant changes in activity across all three body axes—dorsoventral, mediolateral, and longitudinal—beginning at 4 weeks post-application, with no further significant changes observed thereafter. These findings suggest an increase in muscular activation resulting from DME. Additionally, DME appears to elicit a delayed increase in SL at the trot, but not at the walk, starting at 6 weeks, likely as a consequence of enhanced muscular activation. Stride SYM also improved from 6 weeks onward, observed at both walk and trot. Overall, the application of DME led to enhanced dorsoventral, mediolateral, and longitudinal activities, reflecting improvements in elasticity, flexibility, propulsion capacity, stride length, and locomotor symmetry. Importantly, the timing of these adaptations varied over the course of the study, highlighting the progressive nature of the responses.

## Data Availability

The data are available from the authors upon reasonable request.

## Abbreviations

The following abbreviations are used in this manuscript:

DME	Dynamic mobilization exercises
SL	Stride length
DVA	Dorsoventral activity
MLA	Mediolateral activity
LA	Longitudinal activity
REG	Regularity
SYM	Symmetry
FFT	Fast Fourier Transformation
TA	Total activity
DVD	Dorsoventral displacement
SF	Stride frequency
